# Blue-native PAGE in plants: a tool in analysis of protein-protein interactions

**DOI:** 10.1186/1746-4811-1-11

**Published:** 2005-11-16

**Authors:** Holger Eubel, Hans-Peter Braun, A Harvey Millar

**Affiliations:** 1 ARC Centre of Excellence in Plant Energy Biology, University of Western Australia, 35 Stirling Hwy, Crawley 6009, Perth, Australia; 2 Abteilung Angewandte Genetik, Universität Hannover, Herrenhäuser Str. 2, 30419 Hannover, Germany

**Keywords:** Gel-based Proteomics, 2D-PAGE, protein complexes, hydrophobic proteins, Coomassie, solubilization

## Abstract

Intact protein complexes can be separated by apparent molecular mass using a standard polyacrylamide gel electrophoresis system combining mild detergents and the dye Coomassie Blue. Referring to the blue coloured gel and the gentle method of solubilization yielding native and enzymatically active protein complexes, this technique has been named Blue-Native Polyacrylamide Gel-Electrophoresis (BN-PAGE). BN-PAGE has become the method of choice for the investigation of the respiratory protein complexes of the electron transfer chains of a range of organisms, including bacteria, yeasts, animals and plants. It allows the separation in two dimensions of extremely hydrophobic protein sets for analysis and also provides information on their native interactions. In this review we discuss the capabilities of BN-PAGE in proteomics and the wider investigation of protein:protein interactions with a focus on its use and potential in plant science.

## Introduction

With several plant genome sequencing projects already finished and several others nearing completion, the logical next step is the assessment of the protein products encoded by these genomes. The protein composition of different tissues, cell types and isolated cellular organelles from a range of plant species have been investigated to date making use of peptide mass spectrometry and pattern matching to genomic data. These studies have used a variety of approaches to separate proteins, but isoelectric focussing (IEF) followed by SDS-PAGE (typically termed a 2D gel) is the most common technique undertaken. This dominance is despite the fact that this technique is severely limited in its ability to display the very large complement of hydrophobic proteins from plants. Further, even if improvements in this standard 2D gel technique could further alleviate this hydrophobicity problem, a complete understanding of the processes taking place within cells will require much more than the simple identification of the individual polypeptides forming the proteome. Most cellular processes require the action of several enzymes, often each containing multiple subunits. To raise the efficiency, specificity and speed of metabolic pathways, these enzymes are often associated with each other, forming temporary or stable larger protein complexes. Knowledge of the composition and/or structure of these protein complexes will result in a much deeper understanding of metabolic pathways and cellular processes than protein identities alone are able to deliver.

There are many ways to investigate protein interactions, each with individual advantages and drawbacks. Many of the approaches commonly used today are investigative studies of the actual or possible interaction partner(s) of a particular protein of interest. Examples include yeast two-hybrid systems, immunoprecipitation studies with specific antibodies and the more recent use of TAP/FLAG pull-down assays, and FRET/BRET fluorescence studies *in vivo*. However, these approaches are not designed to provide a general overview of protein-protein interaction in a complex proteome of choice by a single experiment.

For an in-depth investigation of the protein complexes forming the respiratory chain of various organisms, Schägger et al. [1] developed a novel experimental strategy to investigate the individual components of this biochemical pathway. Through the combination of mild detergents and the dye Coomassie blue, substituting for the highly denaturating detergent sodium dodecyl sulphate (SDS), it was possible for the first time to separate intact respiratory protein complexes by electrophoresis. Referring to the blue coloured gel and the gentle method of solubilization yielding native and enzymatically active protein complexes, this technique has been named Blue-Native Polyacrylamide Gel-Electrophoresis (BN-PAGE). Over the last 10 years, BN-PAGE in combination with second dimension SDS-PAGE has been the method of choice for the investigation of the respiratory protein complexes of the electron transfer chains of a range of organisms, including bacteria, yeasts, animals and plants. This technique allows the separation in two dimensions of extremely hydrophobic protein sets for analysis and also provides information on their native interactions. There is now a growing number of publications employing this method for the investigation of other hydrophobic and hydrophilic high molecular weight protein complexes in different organisms. Recently, with the introduction of even more sophisticated solubilization methods, BN-PAGE has been used to detect specific interactions between large protein complexes that has led to the discovery of so-called 'supercomplexes'.

In this review we discuss the capabilities of BN-PAGE in proteomics and the wider investigation of protein:protein interactions with a focus on its use and potential in plant science. Major advantages and disadvantages of this technique when compared to other experimental strategies will be mentioned and a short overview about past and possible future applications of BN-PAGE in the plant sciences is provided.

## The working principle of BN-PAGE

### Electrophoretic separation

**Figure 1:**
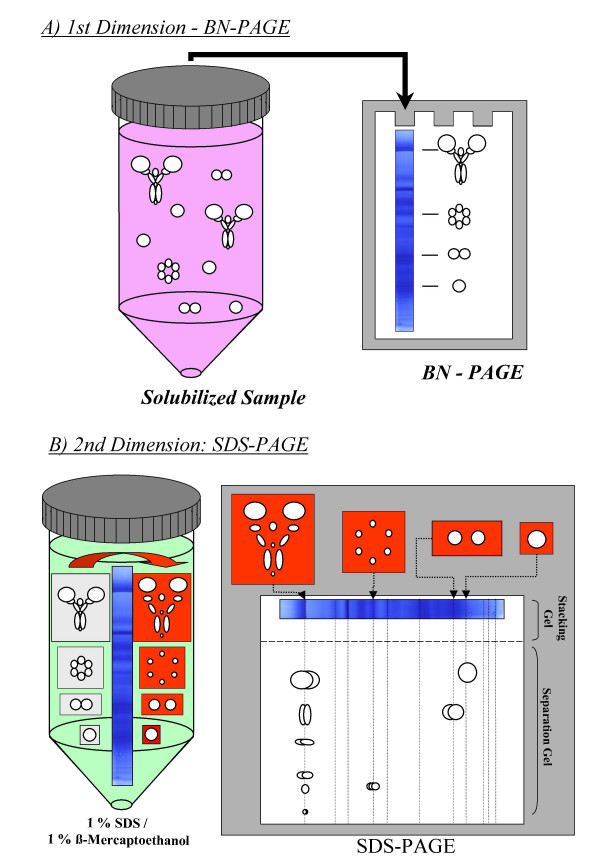
The working principle of BN/SDS-PAGE. A) After solubilization, the mixture of different protein complexes is separated by BN-PAGE, according to their molecular weight. B) Following the gel run, the lane of the gel is excised and subjected to a denaturation solution (eg 1% SDS and 1% β-mercaptoethanol) so that the native protein complexes (underlayed in grey colour) dissociate to their constituent polypeptides. Loosely stuck in the pores of the gel, the subunits of the protein complexes (underlayed in red colour) remain at their position until they are forced electrophoretically into the second dimension gel. Due to the SDS used in the denaturation step (and residual Coomassie) the polypeptides are uniformly negatively charged and are separated according to their molecular weight in the gel. Subunits of a protein complex form a vertical row on the second dimension gel.

In the popular denaturing SDS-PAGE technique, the ionic detergent SDS functions both in solubilizing and denaturing the proteins as well as providing negative charge and therefore unidirectional mobility to the proteins during electrophoresis. In BN-PAGE these multiple functions are accomplished by different reagents. While denaturing is not desired, the solubilization of membrane protein complexes is necessary and is undertaken by mild non-ionic detergents like dodecylmaltoside, Triton X-100 or digitonin. Negative charges are attached to the solubilized protein complexes not by detergents but by the addition and binding of the negative charged dye Coomassie Blue G250. Dissociated dye, separated from the proteins during the gel run is substituted for by a high concentration of Coomassie Blue in the cathode buffer (Figure 1A).

### Preparing the sample

#### (a) Pre-fractionation and enrichment

The reason that BN-PAGE has been most commonly used for the analysis of the respiratory and photosynthetic protein complexes is likely the high abundance of them within the proteomes of these organelles. Relatively little sample material is required for a good visualization of these complexes as more than half of the total protein content of these organelles as estimated to be protein complexes. Low abundance complexes also exist in these organelles, but will remain in the background or may be masked in BN-PAGE separations by the dominant complexes. Successful use of the BN-technique for other applications may require pre-treatment to enrich the protein complex of interest. This could involve a simple concentration step (e.g. by dialysis or ultrafiltration), the removal of unwanted high abundant protein complexes (e.g. by affinity chromatography) or a fractionation step. Fractionation of the sample by a property other than size (e.g. by subcellular separations, ion-exchange chromatography, native IEF) can reduce complexity and therefore compensate for the limited range of sensitivity of BN-PAGE. Prior to the BN-PAGE, it might be necessary to change the buffer system in the sample depending on the method of pre-fractionation used.

#### (b) Solubilization of membrane samples

Compared to IEF, the collection of detergents tested for BN-PAGE is still relatively small. Finding suitable detergents for the solubilization of different protein complexes is a key for a wider application of BN-PAGE in the investigation of membrane protein complexes. A detergent suitable for a native solubilization of protein complexes prior to BN-PAGE must fulfil several prerequisites. It must be as mild as possible but able to disrupt lipid-lipid interactions without disturbing those between protein components in complexes. Disruption of certain lipid-protein associations is usually not desired because it may result in a weakening or even dissociation of protein complexes, especially if lipids are a structural component of the complex. The detergent should also not interfere with the electrophoresis process. Detergents used routinely for the solubilization of protein complexes include *n*-Dodecylmaltoside, Triton X100 and digitonin. Additionally, octylglucoside [2], Brij 96 [3] and saponin [3] have been used (Table 1). Other non-ionic detergents including Big CHAPS, C_12_E_5/8_, *n*-Decanoylsucrose and NP-40 may also be suitable for the native solubilization of membrane protein complexes. Some of these reagents have a defined composition and are synthesized to high degrees of homogeneity, while others such as digitonin are complex mixtures purified from natural sources. Comparative testing of different detergents in various detergent-to-protein ratios in the presence of different salts is necessary to determine the optimal detergent and the right conditions for solubilization [4-6]. A salt of low ionic strength like aminocaproic acid or potassium acetate can further support the solubilization process of membrane complexes.

**Table 1:** Detergents successfully employed for the solubilization of protein complexes prior to BN-PAGE.

*Detergent*	*Sample*	*Detergent concentration*	*Reference*
Dodecylmaltoside	Mammalian	1.1%	[1]
	Mitochondria		
	Plant Mitochondria	1.5%	[103]
	Chloroplasts	2.0%	[75]
	Yeast Peroxisomes	0.5% and 1.0%	[81]

Digitonin	Mammalian	4 g/g protein	[104]
	Mitochondria		
	Plant Mitochondria	5.0 g/g protein	[6]
	Chloroplasts	1.5 g/g protein	[41]
	Yeast Microsomes	1.0%	[80]
	Yeast Peroxisomes	1.0%	[81]

Triton X-100	Mammalian	1.0, 1.4 and 2.4%	[105]
	Mitochondria		
	Plant Mitochondria	0.5%	[6]
	Yeast Peroxisomes	0.2% and 1.0%	[81]
	Whole cellular lysates of different human cell lines	0.1%	[3]

**Brij 96**	Whole cellular lysates of different human cell lines	0.5%	[3]

**Octylglucoside**	Tobacco microsomes	40 mM	[2]

**Saponin**	Whole cellular lysates of different human cell lines	1.0%	[3]

The analysis of mitochondrial respiratory chain complexes utilizing BN-PAGE has shown the influence of the detergent on the results and the conclusions drawn from these results. Classically, n-dodecylmaltoside and Triton X100 have been used exclusively to solubilize the respiratory chain complexes of the inner mitochondrial membrane for BN-PAGE. Using these detergents, only bands representing singular respiratory complexes I, II, III, IV and V can be found on the gels. It was assumed that the complexes found on the gels reflect the situation *in vivo*, supporting the "liquid state model" of individual respiratory protein complexes which are free to laterally diffuse in the inner mitochondrial membrane. However, since the introduction of digitonin for the solubilization of the respiratory protein complexes, the model of the respiratory chain has changed considerably. Stoichiometric association of several components of the electron transfer chain clearly indicate a "solid state model" for the respiratory chain of several organisms. In this model, the protein complexes are not randomly distributed within the inner mitochondrial membrane but form different types of defined arrangements which are called supercomplexes [5]. As the singular respiratory complexes solubilized by DDM and Triton X100 are active, the biological function of the supercomplexes remains unclear, but pictures from electron microscopy strongly support the solid state model [7].

The different membranes within eukaryotic cells each possesses a distinct lipid composition and lipid to protein ratio and different detergents in different concentrations will be needed for the optimal solubilization of their protein complexes. Additionally, plants are able to alter the lipid composition of their membranes to compensate for changes in the climatic conditions like cold stress [8,9]. This effect may also influence the suitability of detergents.

#### (c) Preparation of soluble complexes

Although theoretically less troublesome to investigate than membrane protein complexes, relatively few publications so far use BN-PAGE for the analysis of soluble protein complexes. Direct application of soluble plant cell extracts to BN-gels can result in visible smearing and/or excessive narrowing or widening of the lanes. This is often due to an inappropriate salt concentration for the running conditions of the gel. A buffer exchange to standard BN conditions, for example by dialysis, can help to reduce these symptoms. This, however, has to be undertaken cautiously as soluble protein complexes tend to be more sensitive to disruption by high salt concentrations than membrane complexes and even low salt concentrations may result in a dissociation of soluble proteins [10]. Stability of some protein complexes may also be increased if Coomassie Blue is not added to the sample prior to the gel run. Lengthy exposure in too much Coomassie dye can result in the dissociation of protein complexes. In these cases, the amount of dye in the cathode buffer is usually sufficient for the electrophoresis run [11].

### Maximising the resolution of BN separation

To maximise resolution of the BN-gel, it usually consists of an acrylamide concentration gradient from 3–5% (w/v) at the top (cathodic end) to 13–16% (w/v) at the bottom (anodic end) (Figure 1A). Depending on the acrylamide gradients and their size, protein complexes appear to become lodged at different positions in the gel during the run, resulting in an apparent separation of the complexes according to molecular mass. BN-PAGE was initially invented for the investigation of respiratory chain components, thus it was optimised for the separation of 0.1 to 1 MDa protein complexes. With a reduction of the acrylamide concentration in the separating gel it is possible to resolve complexes of up to 3–4 MDa on BN-PAGE. The use of agarose to replace polyacrylamide in the separating gel can be considered if even larger complexes or supercomplexes (up to ~10 MDa) are expected [10].

### Second and third dimensions for BN gels

In combination with an SDS gel system as second dimension, BN-PAGE is often used as the first dimension of multiple dimension gels (Figure 1B). For a second dimension, a lane of the BN-gel can be cut out and placed horizontally on the second dimension gel (that is at 90° to the direction of the first electrophoretic separation). To aid transfer of the protein complexes/proteins into the second dimension gel, the first dimension lane can be placed between the glass plates of the second dimension gel on its initial assembly. The gel is then cast with the lane already in position and it is later completely embedded in the stacking gel, thus ensuring a smooth transition of the proteins from the first into the second dimension. In this case it is useful to make the second dimension gel thinner (e.g. 1 mm) than the first dimension (e.g. 1.5 mm) to ensure that the BN gel strip stays in place during this procedure. A stacking gel is necessary for focussing of the now vertical bands of the BN gel lane. Depending on the type of second dimension used, the BN-gel lane can be treated prior to assembly of the second dimensions with different reagents (see below). BN-PAGE can also be combined with IEF/SDS-PAGE, resulting in a 3D gel system allowing very high resolution of protein samples (see below).

#### BN/SDS-PAGE

**Figure 2:**
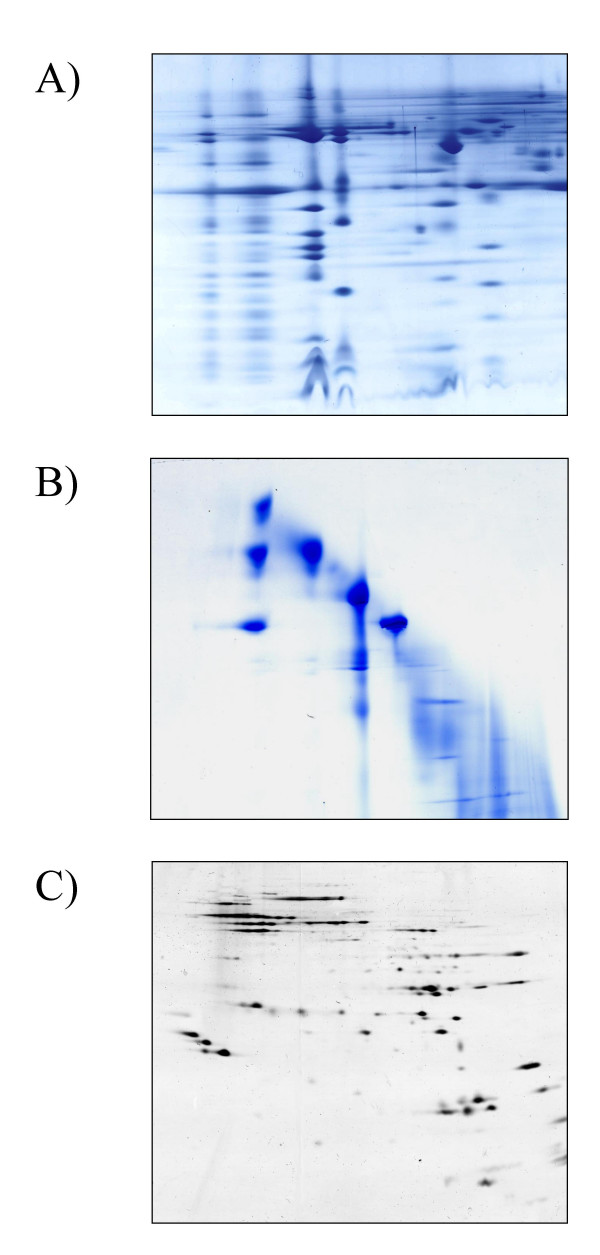
Second and third dimensions for BN-PAGE of Arabidopsis mitochondrial protein complexes. A) BN-PAGE of a digitonin-solubilized fraction followed by denaturing and reducing SDS-PAGE (BN/SDS-PAGE); B) BN-PAGE of Digitonin-solubilized protein complexes followed by a second BN-PAGE (BN/BN-PAGE) employing n-Dodecylmaltoside to introduce slightly less native conditions inducing partial dissociation of protein supercomplexes; C) IEF/SDS-PAGE of the ATP-Synthase band of a dodecylmaltoside-solubilized sample. The band has been cut out of the gel and the proteins have been subsequently electroeluted under denaturing conditions followed by a precipitation step to concentrate and purify the proteins for isoelectric focussing. The image (c) was reproduced from figure 1 in [15] with permission from Wiley-VCH.

The BN gel strip should be incubated prior to the casting of the second dimension in a buffer containing SDS and 2-mercaptoethanol (Figure 1B). This step ensures complete denaturation of the protein complexes necessary for the subsequent separation of their subunits. The stacking gel is cast around the first dimension BN gel lane and preferably uses the same gel buffer. For visualisation and separation of samples containing small proteins of interest (<15 kDa) best results are obtained if the second dimension gel is a Tricine system consisting of a stacking, spacer and separating gel [12]. Good results can also be obtained using a standard Glycine based system [13] when analysis is concentrating on larger proteins (>15 kDa). A typical BN/SDS-PAGE from a plant mitochondrial membrane sample is shown in Figure 2A.

#### BN/BN-PAGE

Different non-ionic detergents appear to alter the stability of protein complexes. For example, digitonin can be used for the solubilization of protein complexes and it has been shown to allow stabilization of supercomplexes in particular samples, while dodecylmaltoside destablizes some of these complex interactions [6,7]. Treatment of a first dimension BN-lane of digitonin-solubilized supercomplexes with DDM leads to the dissociation of these structures into single protein complexes. On the resulting BN/BN gels, intact supercomplexes and protein complexes form a diagonal line, whereas those which are specifically destabilized under the conditions of the second gel dimension migrate below the diagonal (Figure 2B). Alternatively to such detergent treatments, the BN strip of the first gel dimension can also be subjected to other mildly denaturing conditions like elevated temperature, salt concentration, reductants or other suitable chemicals. By comparison of the electrophoretic mobility of the decomposition products with that of the intact singular complexes on BN/BN gels, the protein complexes forming the larger supercomplexes can be identified. This can be confirmed by mass spectrometry of the complex components. If BN/BN gels are intended to be used to perform *in-gel *activity stains, it can be necessary to cast the second dimension gel first and, after complete polymerization, place the first dimension lane on top of it. This avoids contact of the complexes with highly reactive APS and TEMED and can help to maintain enzymatic activities [14].

#### BN/IEF/Tricine-SDS-PAGE

BN gels can also be used to reduce the complexity of a sample for subsequent IEF/SDS-PAGE. A band is cut from a BN gel and the proteins then electroeluted. A standard 2D-IEF/SDS-PAGE can be performed on this protein sample subsequently, separating only the subunits of the protein complex of choice [15] (Figure 2C). This so-called 3D gel system has the advantage of a higher resolution power over normal BN/SDS-PAGE as the polypeptide subunits are resolved by IEF and SDS-PAGE dimensions. It notably allows the separation of isoforms of subunits of the same molecular weight if they differ in their isoelectric points.

### Staining BN gels

**Figure 3:**
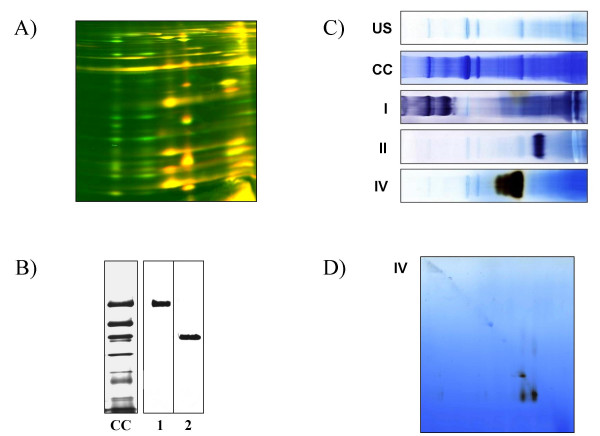
Differential gel electrophoresis (DIGE) stains, immuno-stains and in-gel activity stains of protein complexes separated by BN-PAGE. A) DIGE staining of mitochondrial protein complexes on BN-SDS gels. Proteins displayed in red are from an Arabidopsis mutant with a reduced abundance of complex I, green proteins are subunits of the wild-type sample. If mutant and wild-type proteins are present in the same amount, they are coloured yellow. This image was reproduced from Figure 8a in 25 with permission from Elsevier. B) Immunoblot of two lanes of a BN-gel and a Coomassie-colloidal stained reference lane (CC). Lane 1 has been probed with an antibody directed against respiratory complex I, lane two with an antibody against complex III. This image was reproduced from Figure 2 in [103] with permission from Blackwell Publishing. C) In-gel activity stains of Arabidopsis respiratory complexes I, II and IV. An unstained (US) and Coomassie colloidal (CC) stained gel lane are given as references. This image was reproduced from Figure 2 in [35] with permission from Elsevier. D) Complex IV activity stained BN/BN gel of the same sample.

After thorough removal of the Coomassie dye in a solution of 40–50% methanol and 10% acetic acid, first dimension BN-gels can be stained with all commonly applied procedures like silver stains, classical and colloidal Coomassie stains and fluorescent dyes. Differential staining procedures using covalently bound fluorescent dyes of different colours (Differential gel electrophoresis, DIGE) have also been employed successfully on BN gels [16] (Figure 3A). It is, however, important to use fluorescent dyes that covalently bind to lysine residues rather than cysteine residues, as the latter may tend to destabilize disulphide linkages between and within protein complexes prior to BN separation which makes the sample unsuitable for analysis of native complex structure (H. Eubel, unpublished results).

### Western blotting of BN gels

First dimension BN gels can be successfully electroblotted onto nitrocellulose or PVDF membranes if a few requirements are met (Figure 3B). Firstly the gel run has to aborted at an early stage (i.e. after half to two third of the normal run time) to prevent the complexes from becoming stuck in the pores of the gel. Secondly, due to the high mobility of Coomassie and its high concentration in the gel, the cathode buffer should be exchanged for one containing no Coomassie after approximately two hours of the run to prevent excessive dye deposition on the membrane during transfer [11]. Alternatively, the membrane can be replaced a few times during the early stages of the blotting process to remove the bulk of the Coomassie dye. PVDF membranes generally have a lower affinity for Coomassie than nitrocellulose [11].

### Identification of protein complexes

If a reference gel of the same sample type from a different species exists, protein complexes can often be identified by a simple comparison of the subunit clusters on BN/SDS gels. Within a kingdom, orthologous protein complexes often have similar amount of subunits with a similar distribution of molecular masses. This gives a good first hint of their identity. Because of evolutionary distance, a comparison between species of different kingdoms is often difficult. The most effective way to identify unknown protein complexes is mass spectrometry. Samples directly cut out of a first dimension BN gel or a BN/BN gel can be subjected to complex mixture tandem mass spectrometry to identify a series of proteins in the complex. Alternatively, individual subunits derived from a BN/SDS PAGE gel can be used for identification by tandem mass spectrometry or peptide mass fingerprinting. Often, the identification of a few subunits is sufficient for a reasonable identification of the complex. In a complementary strategy to consider complex function and also to aid identification, colorimetric enzyme assays can be performed as in-gel activity stains of first dimension BN [17,18] (Figure 3C) or second dimension BN/BN gels [14] (Figure 3D).

## Known limitations of BN-PAGE

### Detergents

For membrane protein complexes, the use of BN-PAGE is only limited by the availability of a suitable detergent for the native solubilization of the protein complexes. However, to date, only a very restricted collection of detergents has been tested for this application and, unfortunately, so far there seems to be no single detergent which is suitable for solubilization prior to BN-PAGE for all protein complexes of interest (Table 1). More extensive testing of detergents is required to determine the real limitations of the BN technique for the analysis of membrane protein complexes.

### Resolution

The number of spots from a sample resolved on a 2D BN/SDS PAGE gel is normally smaller than from the same sample resolved by 2D IEF/SDS PAGE. This is mainly due to the fact that proteins are not distributed over the entire 2D area in BN/SDS PAGE, but arranged in vertical rows. Even more than in other gel based proteomic approaches, this dictates a reduction in sample complexity for BN separations. Single proteins or complexes of a molecular weight <100 kDa are not well resolved in BN-PAGE due to the high abundance of proteins in that size range and the limited separation distance resulting from the acrylamide gradient. Another resolution drawback seems to be a rather limited dynamic range, leading to a focus on the most prominent protein complexes and an under-representation of lower abundant complexes on BN gels.

### Artefacts

Co-migration of proteins on BN-gels is not a final proof of native association as physically distinct complexes may have similar molecular masses and therefore may appear in the same protein bands. However, the resolution of this technique is much higher than conventional gel filtration chromatography approaches, and if the sample has been well fractionated to limit complexity we consider it an excellent line of evidence for association. Confirmation can be obtained using immunoprecipitations or co-migration under different conditions (eg non-sized-based chromatography). Weak or transient interactions between proteins or protein complexes constitute another challenge to the system. Even if an appropriate detergent is found, it inevitably will weaken the association of the proteins. The addition of Coomassie might lead to dissociation of fragile protein complexes, because negative charges on protein subunits of a protein complex can lead to electrical repulsion. The binding capacity of Coomassie and therefore the electrophoretic mobility depends on the physical and chemical properties of the proteins, e.g. size, shape, hydrophobicity, post-transcriptional modifications and isoelectric point. This can make it hard to judge the precise molecular mass of a band on a BN-gel [19].

## Current biological applications of BN-PAGE

### Studies of mitochondrial and chloroplast electron transport chains

BN-PAGE has been extensively used to investigate the structure of the respiratory chain in plants. In mammalian mitochondrial samples all components of the OXPHOS protein complexes could be resolved in the initial investigations using BN-PAGE [1]. However, in plant mitochondria only complexes I, III and V were present on the first BN-PAGE gels and dissociation of particular complexes was noted such as the release of the F1 component of the complex V [20]. The assembly of complex I of maize mitochondria [21] as well as *Chlamydomonas *has been studied by BN-PAGE [22]. Also, the composition of complex I and function of plant specific subunits in Arabidopsis have been investigated [23-25]. Resolution of complex III has allowed investigation of the core proteins of cytochrome reductase from potato [20] and the co-evolution of these proteins in different organisms [26]. Proteins responsible for the development of cytoplasmic male sterility (CMS) have been identified by BN-PAGE as being subunits of complex V [27,28]. BN-PAGE has also been employed to monitor the purity of mitochondria isolations from *Brassica *[29] and *Arabidopsis *[30,31]. Since the completion of the *Arabidopsis *and rice genome sequencing project and the onset of gel based proteomics, BN-PAGE has also been employed for more complete investigations of the respiratory chain [15,32], aiming to provide a broader overview of plant mitochondrial protein complexes and their subunit composition. However, complexes II and IV were still missing on most of these gels. This changed with the introduction of digitonin for the solubilization of mitochondrial membrane protein complexes. Digitonin not only led to the stabilisation of these other complexes on the gels and the discovery of plant specific subunits within them [6,33], but it also prevented the dissociation of respiratory supercomplexes, formed by components of the electron transfer chain [6,14,34,35].

Several studies have also used BN-PAGE for the analysis of photosynthetic protein complexes and the plastidic F_0_F_1-_ATPase [36-50]. Again, the utilization of digitonin has facilitated the solubilization of protein supercomplexes [41], confirming results obtained by crystallography and electron microscopy followed by single particle analysis (for a review see [51]). Other research utilizing this technique in plant organelle research includes the investigation of the NAD(P)H dehydrogenase complex of thylakoid and etioplast membranes [52-56].

### Other applications in plants

In a number of publications BN-PAGE has also been used for the survey of the mitochondrial and plastidic protein import apparatus [15,57-62] and the thylakoid protein insertion systems [63-66]. It has been employed for the characterization of the tobacco plastid-encoded plastid RNA-polymerase complex [67,68] and plastidic omega-3 saturases [69] as well as a 350 kDa plastid ClpP protease complex [67]. BN-PAGE has also been used for the analysis of the cytochrome c maturation complex [70], an acetyl-coenzyme A carboxylase [71], an isovaleryl coenzyme A dehydrogenase [72], the formate dehydrogenase complex [73] and several other soluble mitochondrial protein complexes [74]. Protein complexes of the plasma membrane of spinach have been successfully resolved by BN-PAGE [75] as have complexes in the peribacteroid membrane from *Lotus japanicus *root nodules [76] and in the microsomes of *Arabidopsis *[77]. A ~200 kDa protein complex from peanut representing a putative food allergen has also been characterised by BN-PAGE [78].

### Diverse applications in bacteria, yeast and mammals

Using several different detergents and antibodies, Camancho-Carvajal et al. [3] identified several protein complexes in whole cell lysates of human cell lines without previous subfractionation and enrichment. Applying BN-PAGE and other techniques, it was also found that the nucleosome assembly protein NAP-2 forms part of multiprotein complexes in human HeLa cell cultures [79]. Shibatani et al. [80] discovered that a mammalian oligasaccharyltransferase complex forms an integral component of the ER translocation machinery. More than ten protein complexes were discovered in the peroxisomal membrane of the yeast *H. polymorpha *[81]. In several publications, BN-PAGE has been used to investigate presenelin/γ-secretase complexes, associated with Alzheimer's desease [82-87]. Krall et al. resolved different VirB protein complexes of *A. tumefaciens *[88]. The *E. coli *Twin Arginine Translocase [89] has been analysed by BN-PAGE as well as the human glycine receptor, a member of the ligand-gated ion channel (LGIC) superfamily I, expressed in *Xenopus *oocytes [90]. Recently, a systematic analysis of the *E. coli *envelope revealed the presence of 43 protein complexes [91], thus making it the most comprehensive approach to membrane protein complex analysis employing BN-PAGE to date. As a result of its broad analysis, this study was also able to assign several proteins with unknown functions to specific protein complexes.

## Future opportunities for BN in plant research

It is becoming more and more apparent that protein complexes are not an exception, but are a basic working principle of the cell. BN-PAGE can provide data not only on protein complex composition, it can also be used to increase our knowledge of the protein content of a proteome of choice. The use of BN-PAGE in the investigation of plant membrane complexes has already revealed numerous differences in the plant metabolism when compared to that of bacteria, fungi & mammals. Taking the lifestyle of plants into consideration, a large number of different or unique metabolic pathways might be expected to be present. These pathways almost certainly demand the existence of plant specific protein complexes. So far, most protein complexes characterised to date in plants are involved in energy metabolism and the genetic replication system. The large number of different pores, transporters and carriers present in the membranes of plant cells are still not characterized at a biochemical level, the same is true for the many protein complexes involved in signal perception and signal transduction. Due to its relatively lower resolution, when compared to other gel based techniques, the future of BN-PAGE will likely be linked to the investigation of defined subproteomes of the cell.

### BN-PAGE to further explore plant proteomes

Approaches to separate and analyse proteomes will continue to be developed to complement IEF-SDS/PAGE. The combination of techniques will most probably result in a higher coverage of any given proteome. On one hand non-gel approaches are becoming a dominant approach to separate and analyse peptides from complex mixtures by mass spectrometry [92,93]. These have the advantage of better representing the hydrophobic proteome, but suffer from losses in the quantification of members of the proteome compared to IEF-SDS/PAGE. On the other hand, an approach like BN-PAGE can maintain quantification and greatly improve hydrophobic protein identification. A direct comparison of the protein sets of the Arabidopsis mitochondria proteome derived either by IEF/SDS-PAGE [30,94] or BN/SDS-PAGE [6,23,33] reveals only a slight difference in the average of the Grand Average of Hydropathicity (GRAVY) scores of the sets (-0.23 and -0.13). The root mean square, however, is two times higher for the BN derived-set (0.40) than for the IEF derived-set (0.20). This clearly indicates a much broader range in the GRAVY scores for the set derived from BN/SDS-PAGE. In the BN set, 22% of proteins possess GRAVY scores above zero, whereas this is only the case for 7% of proteins identified by IEF.

It might be argued that the technical aspects of BN-PAGE make it unlikely to be widely adopted outside specialist laboratories. To date, users of BN-PAGE still have to prepare their own first dimension gels. Being an acrylamide gradient, it can be compared to preparing IEF tube gels in terms of labour and time requirements. However, in contrast to IEF, there is no special equipment needed to perform BN-PAGE. Any common vertical gel system can easily be modified to suit the requirements for BN-PAGE. The original protocol for IEF/SDS-PAGE [95,96] used self cast, labour intensive tube gels for the first dimension. Since the introduction of commercially available IEF systems employing immobilized pH-gradient (IPG) strips, the use of tube gels has greatly reduced. Handling of the IPG strips is easy and, because of mass-production, they ensure a higher reproducibility. Several kinds of IPG strips are commercially available, giving the researcher the opportunity to choose the appropriate pH range and gradient for his application. More commercial products for BN could be produced if the market expanded.

### BN-PAGE for the analysis of protein:protein interactions in plants

Most proteome analyses in plants to date either considers the location of proteins within cells or are a differential analysis of expression in response to development, environment or disease. This lacks the information about the interaction pattern of these proteins, which is a very important aspect of understanding the processes carried out by the members of this proteome. Popular techniques to determine the interaction partners of proteins are the yeast-two-hybrid technique (Y2H), affinity chromatography (eg tandem affinity purification [TAP]), fluorescence resonance energy transfer (FRET), bioluminescence resonance energy transfer (BRET), bimolecular fluorescent complementation, high-throughput mass spectrometric protein complex identification (HMS-PCI), size exclusion chromatography (SEC) and cross-linking experiments. Most of these techniques require the organism of choice to be accessible to genetic engineering. With the exception of SEC and some cross-linking techniques, all these approaches have in common that they focus on a single protein and determine its binding partners.

The yeast two-hybrid system has been used extensively to study protein:protein interactions in *S. cerevisae *[97,98] but is known to produce a relatively high number of false positive results [98]. In one of the largest uses of HMS-PCI, 493 bait proteins in *S. cerevisae *were found to be involved in 3617 interactions after correction for false positives, 74% of these interactions could be confirmed by immuno-precipitation experiments [99]. These numbers give insight into the complexity of protein networks within a cell and emphasize the need for complementary ways for the analysis of in vivo protein interactions. Being a preparative technique, SEC has a lower resolution compared to BN-PAGE. Also, the micelle size of the detergent and possible unspecific aggregations have an influence on the apparent size of a protein complex in SEC. In BN-PAGE, these effects are reduced by the presence of Coomassie blue and aminocaproic acid [2]. Chemical crosslinkers are usually only suitable for the analysis of small protein complexes and cross-linking experiments often suffer from poor yields of the interacting proteins [100]. This often necessitates long incubation times that in turn increase the probability of the generation of artefacts or leading to a destabilization of the complex. With the introduction of photo-inducible cross-linking of proteins, the risk of artefact-formation has been reduced considerably [101]. The introduction of cleavable cross-linkers allows the direct identification of protein interaction sites by mass spectrometry [102]. However, to our knowledge, this technique is still limited to relatively small protein complexes. It also allows no rating of the binding strength between the proteins within the complex due to the covalent modifications introduced by the technique. And of course, great care has to be taken in the choice of the cross-linking reagent to avoid false positive results. BN offers a broader approach as the composition of many small and large protein complexes and therefore the interaction patterns of all their protein-subunits in a relatively complex sample can be determined in a single experiment. Further, protein overexpression and/or chemical modification is not involved, which often allows high yields of native complexes for analysis.

## Conclusion

Since its invention, BN-PAGE has had an enormous impact on the investigation of the respiratory chains and photosynthetic complexes in a range of organisms. When used in a 2D system, BN-PAGE allows an assignment of proteins to their protein complexes and display of highly hydrophobic proteins in two dimensions. Recently, BN-PAGE is beginning to be applied to different types of samples and with many different aims. These range from the assessment of the oligomeric state of protein complexes to the analysis of complex mixtures of protein complexes. BN-PAGE, together with other techniques, might be the ideal tool to begin study of new protein complexes in plants and gain a deeper understanding of the unique aspects of protein-protein interactions in plant cellular processes.

## Competing interests

The author(s) declare that they have no competing interests.
